# Exosomes derived from human adipose mesenchymal stem cells attenuate hypertrophic scar fibrosis by miR-192-5p/IL-17RA/Smad axis

**DOI:** 10.1186/s13287-021-02290-0

**Published:** 2021-03-31

**Authors:** Yan Li, Jian Zhang, Jihong Shi, Kaituo Liu, Xujie Wang, Yanhui Jia, Ting He, Kuo Shen, Yunchuan Wang, Jiaqi Liu, Wei Zhang, Tongtao Wang, Zhao Zheng, Dahai Hu

**Affiliations:** 1 Department of Burns and Cutaneous Surgery, Xijing Hospital, Fourth Military Medical University, 127 West Chang-le Road, Xi’an, 710032 Shaanxi China; 2 Department of Plastics and Aesthetic Surgery, The First Affiliated Hospital of Xi′an Medical University, No.48 West Fenghao Road, Xi’an, 710077 Shaanxi China

**Keywords:** ADSC-Exo, miR-192-5p, IL-17RA, Smad pathway, Hypertrophic scar

## Abstract

**Background:**

Hypertrophic scar (HS) is a fibro-proliferative disorder of dermis after burn or trauma and usually leads to esthetic disfiguration and functionary impairment for patients. Emerging evidences demonstrated ADSC-Exo could alleviate the visceral fibrosis, but little attention had been paid to its role in skin fibrosis. In the study, we would explore the effect of ADSC-Exo on HS and investigated the exact mechanism underlying the properties.

**Methods:**

ADSC-Exo were isolated, identified, and internalized by HS-derived fibroblasts (HSFs). The effect of ADSC-Exo on the proliferation and migration of HSFs were detected by flow cytometry and Ki67 immunofluorescence staining, or scratch and trans-wells assays, respectively. RT-PCR, immunoblotting, immunofluorescence, and immunohistochemistry staining were used to evaluate the expression of IL-17RA, Col1, Col3, α-SMA, SIP1, and p-Smad2/p-Smad3 in HSFs stimulated with ADSC-Exo, miR-192-5p mimics, or inhibitors, IL-17RA siRNA and their negative controls. Digital morphology, H&E, Masson’s trichrome staining, and immunohistochemistry staining were performed to measure the effect of ADSC-Exo and Lv-IL-17RA shRNA on excisional wound of BALB/c mice.

**Results:**

The verified ADSC-Exo effectively inhibited the proliferation and migration of HSFs, decreased the expression of Col1, Col3, α-SMA, IL-17RA, and p-Smad2/p-Smad3 and increased the levels of SIP1 in HSFs. Besides, the mice in ADSC-Exo-treated group demonstrated faster wound healing and less collagen deposition. Furthermore, miR-192-5p was highly expressed in ADSC-Exo and ADSC-Exosomal miR-192-5p ameliorated hypertrophic scar fibrosis. Meanwhile, miR-192-5p targeted the expression of IL-17RA to decrease the pro-fibrotic proteins levels. Moreover, IL-17RA was overexpressed in HS and HSFs, and knockdown IL-17RA alleviated the expression of Col1, Col3, α-SMA, and p-Smad2/p-Smad3 and increased the expression of SIP1 in HSFs. Most importantly, IL-17RA silence also facilitated wound healing, attenuated collagen production, and modulated Smad pathway in HSFs.

**Conclusions:**

This study illustrated ADSC-Exo attenuated the deposition of collagen, the trans-differentiation of fibroblasts-to-myofibroblasts, and the formation of hypertrophic scar by in vitro and in vivo experiments. ADSC-Exosomal miR-192-5p targeted IL-17RA to regulate Smad pathway in hypertrophic scar fibrosis. ADSC-Exo could be a promising therapeutic strategy for clinical treatment of hypertrophic scar and the anti-fibrotic properties could be achieved by miR-192-5p/IL-17RA/Smad axis.

**Supplementary Information:**

The online version contains supplementary material available at 10.1186/s13287-021-02290-0.

## Background

Hypertrophic scar (HS), which generally develops after severe burn injury or skin trauma, is a fibro-proliferative disorder of cutaneous wound healing that manifests as myofibroblasts activity and collagen deposition. The trans-differentiation of fibroblasts to myofibroblasts is a critical procedure in the pathogenesis of scar formation, which is characterized by alpha smooth muscle actin-positive (α-SMA+) fibroblasts that could stimulate collagen synthesis, particularly Col1 and Col3 [1]. HS causes esthetic destruction and functional impairment, resulting in the physiological and psychological problems [2]. However, the current prophylactic and therapeutic strategies of HS are unavailable. Therefore, it is necessary to explore the novel clinical schedules.

Adipose tissue-derived mesenchymal stem cells (ADSCs) have been widely used as a curative approach in fibrotic diseases because of more extensive distribution and lower immunogenicity [3]. The anti-fibrotic properties of ADSCs were attributed to the paracrine mechanisms [4]. Exosomes, which are secreted from ADSCs and extracted from ADSCs conditioned medium (ADSC-CM), could ameliorate cardiac, liver, and pulmonary fibrosis. It had been reported that ADSC-Exo inhibited the bioactivity of keloid fibroblasts [5–8]. However, little attention had been paid to its role in alleviating hypertrophic scar fibrosis and the underlying mechanism had not been fully understood. Exosomes are natural carrier systems to exert cell-to-cell communication and transport the genetic information (mRNAs, miRNAs, proteins, and lipids) between donor and recipient cells [9]. Growing evidences had been demonstrated exosome-enriched microRNAs played the crucial role in the pathogenesis of visceral fibrosis and tissue regeneration [10–12]. Although the effect of miR-192-5p on fibrotic diseases was controversial, some investigators found miR-192-5p exerted the anti-fibrotic properties in renal fibrosis [13, 14]. In the study, we mainly focused on the roles of ADSC-Exo-derived miR-192-5p in hypertrophic scar fibrosis.

MicroRNAs, endogenous and small noncoding RNAs (18–25 nucleotides in length), could regulate genes expression by inducing the degradation or translational inhibition of targeted mRNA. As an epigenetic regulator, miRNAs were closely associated with the process of skin fibrosis [15]. MiR-192-5p was firstly cloned by Lagos-Quintana et al. [16] and further confirmed by Lim et.al [17]. Together with miR-194, human miR-194-2-192 clusters are co-transcribed at 11q13.1 and have the same seed sequence [18, 19]. The predicting result obtained by bioinformatic analysis indicated there was the binding sites of complementary pairs between IL-17RA 3′UTR and miR-192-5p. As the receptor of IL-17A, IL-17RA, which was initially identified in 1995 [20], had been reported to mainly express in fibroblasts, epithelial cells, smooth muscle cells, and microvascular endothelial cells [21, 22]. In previous literatures, IL-17RA deficiency or blockade was confirmed to inhibit the visceral fibrosis [23–25], but the effect of IL-17RA on scar formation had not been elucidated. Since Smad pathway has been recognized as an important mediator in the fibrotic diseases, by which whether IL-17RA could regulate hypertrophic scar fibrosis? IL-17A/IL-17RA axis was necessary for the production of TGF-β, which was known to activate the phosphorylation of Smad2 and Smad3. Then, phosphorylation of Smad3 was constitutively increased in systemic sclerosis derived fibroblasts [26], and Smad3-deficient mice represented the decrease of collagen deposition compared to wild-type mice when given the treatment of kidney injury [27]*.* As noted above, we hypothesized the anti-fibrotic effect of ADSC-Exo was achieved by miR-192-5p/IL-17RA/Smad axis.

In the study, we identified ADSCs and ADSC-derived exosome (ADSC-Exo), explored the effect of ADSC-Exo on hypertrophic scar fibrosis through in vitro and in vivo experiments, and then investigated the possible mechanism involved with the anti-fibrotic properties of ADSC-Exo*.* The in vitro experiments demonstrated ADSC-Exo could inhibit the proliferation, migration, and contraction of HS-derived fibroblasts (HSFs) and decrease the expression of collagen and α-SMA in HSFs. Simultaneously, the in vivo experiments showed ADSC-Exo facilitated wound healing and attenuated collagen deposition and myofibroblast trans-differentiation in the excisional model of BALB/c mice. Furthermore, we provided the evidences that miR-192-5p in ADSC-Exo attenuated hypertrophic scar fibrosis and miR-192-5p targeted IL-17RA to regulate Smad signal transduction pathway in scar formation. In a word, our work might provide a reasonable explanation for the therapeutic strategy of ADSC-Exo in hypertrophic scar.

## Methods

### Patients and ethics approval

Adipose tissues, hypertrophic scar (HS) tissues, and adjacent full-thickness normal skin (NS) tissues were collected from patients (mean age of 30 years) who underwent plastic excision in our department (Xi′an, China). Before surgery, all patients were informed of the purpose and procedures of the study and agreed to offer their excised tissues. Written informed consent was obtained from all the participants involved in the experiment, and this study was approved by the Ethics Committee of Xijing Hospital affiliated with Fourth Military Medical University.

### The isolation and culture of HSFs

Briefly, the dermal portions of hypertrophic scar tissues were minced and cultured by tissue block explant to isolate HS-derived fibroblasts (HSFs). HSFs were cultured with DMEM (Gibco, Grand Island, NY, USA) supplemented with 10% FBS (Corning, USA), 100 U/ml penicillin, and 100 μg/ml streptomycin in a humidified incubator containing 5% (v/v) CO_2_ at 37 °C. Fibroblasts between the third and fifth sub-passages were used for the following experiments. HSFs were cultured in six-well plates at a concentration of 2 × 10^5^ cells/well, starved in serum-free medium overnight when grown to 70–80% confluent, and then stimulated with ADSC-Exo (20 μg/ml), miR-192 mimics (100 nM), inhibitors (100 nM) and negative control (100 nM), and scramble siRNA or IL-17RA siRNA (100 nM) that transfected with Lipofectamine®2000 reagent (Life Technologies Invitrogen, Carlsbad, CA, USA) for approximately 24 h or 48 h to detect the mRNA or protein levels. The lysates were used to analyze the expression of fibrotic markers (Col1, Col3, and α-SMA).

### The isolation and identification of human-derived ADSCs

As previously reported [28], human subcutaneous adipose tissues were minced and digested with 1 mg/ml type I collagenase (Gibco, Grand Island, USA, Cat.17100-017) in DMEM at 37 °C. Subsequently, the mixers were filtered, centrifuged, and resuspended in human ADSC Expansion Media (OriCell medium, Cyagen, China). ADSCs at 3–5 passages were incubated with fluorescence-conjugated antibodies (CD29-FITC, CD44-PE, CD73-FITC, CD90-FITC, CD34-PE, CD45-FITC) and analyzed by a flow cytometer (BD FACSAria™ III system; BD Pharmingen). For adipogenic and osteogenic differentiation, approximately 80–90% confluent ADSCs were grown in six-well cell culture plates precoated with a 0.1% gelatin solution (Cyagen Bioscience, Inc., Guangzhou, China). Then, ADSCs were incubated with adipogenic differentiation induction medium for 2 weeks or osteogenic differentiation induction medium for 3 weeks, respectively. ADSCs induced by adipogenic and osteogenic differentiation were fixed with 4% paraformaldehyde and stained with Oil Red O or Alizarin Red S to detect the results of inducement culture. Images were observed under an Olympus IX71 light microscope (Tokyo, Japan).

### The isolation, identification, and label of ADSC-derived exosome (ADSC-Exo)

Cell-conditioned medium was collected from approximately 90% confluent adipose tissues derived mesenchymal stem cells (ADSCs or ADSCs transfected with miR-192-5p NC and inhibitor) grown in 100-mm cell culture dishes with human ADSC basal medium containing FBS depleted of bovine serum extracellular vesicles by 16 h ultracentrifugation at 100,000×*g*. Exosomes were isolated from the collected medium by differential ultracentrifugation [29]. All centrifugation steps were performed at 4 °C. The supernatants were firstly subjected to a centrifugation step of 300×*g* for 10 min to pellet and removed cells. Next, the supernatant was spun at 2000×*g* for 10 min to remove debris and apoptotic bodies. Then, the supernatant was centrifuged at 10,000×*g* for 30 min, followed by ultracentrifugation at 100,000×*g* for 70 min using a Ti70 rotor (Optima XPN-100 Ultracentrifuge, Beckman Coulter, Kraemer Boulevard Brea, USA). The pelleted exosome was resuspended in 200 μl PBS, the morphology of isolated exosome was immediately visualized by transmission electron microscope (TEM), and the distribution of size was analyzed by nanoparticle tracking analysis (NTA; ZetaView®system). Meanwhile, immunoblotting was performed to detect the expression of known exosomal markers CD9 and CD63. Next, the concentration of exosomal protein was measured by a BCA protein assay kit and the average level of concentration was adjusted to 2 μg/μl. Exosome diluted in culture medium was passed through a 0.22-μm filter to keep sterilized before the experiment started. The purified exosome was labeled with red fluorescence dye PKH26 to examine the internalization in HSFs (Sigma-Aldrich, St. Louis, USA). Briefly, 250 μl exosome diluted in PBS were incubated with a final PKH26 concentration of 1 × 10^− 6^ M for 5 min, excess dye was neutralized with 1 ml exosome-depleted FBS, the mixture were then ultracentrifuged for 70 min at 4 °C, 100,000×*g* to remove the supernatant and the pellets were resuspended in PBS. HSFs stimulated with PKH26 labeled-exosome in serum-depleted medium for 24 h were fixed with 4% paraformaldehyde. Cells were washed with PBS three times and nuclear was counterstained with DAPI, and the images were observed by FSX100 (Olympus, Tokyo, Japan).

### Real-time quantitative polymerase chain reaction (qRT-PCR)

The samples were lysed with TRIzol Reagent (Takara, Japan), and total RNA was extracted and quantified to confirm the concentration. In total, 500 ng of RNA was reversely transcribed into cDNA using Prime Script™ RT reagent kit (Takara, Japan). The cDNA was amplified with Ultra SYBR Mixture (CWBIO, Beijing, China) and specific primers by Bio-Rad IQ5 Real-Time System (Bio-Rad, Hercules, CA, USA). Reaction mixtures were treated with pre-denaturation at 95 °C for 10 min, amplified for 40 cycles of denaturation at 95 °C for 15 s, and annealed at 60 °C for 1 min followed by melting curve stage. The relative expression was calculated using 2^−ΔΔCT^ method. Each reaction was performed in triplicate to determine the expression of target genes, which were normalized against GAPDH. For miRNA, 800 ng of RNA was used to transcribe for cDNA with a reverse transcription kit supplied by Clontech (Mir-X™ miRNA First-Strand Synthesis). RT-PCR was performed with miScript SYBR green PCR kit and miRNA-specific primers, and U6 was recognized as an internal control. All PCR experiments were performed in triplicate. The primer pairs used in the study were listed in Table 1 of supplementary materials.

### Western blotting

To extract cellular and tissular proteins, fibroblasts and skin tissues were collected, washed twice with ice-cold PBS, and solubilized in cell lysis buffer (RIPA, Beyotime) supplemented with proteinase inhibitor (PMSF, Boster, China). Lysed samples were incubated on ice for 30 min. Cell lysates were then centrifuged at 12000 rpm at 4 °C to remove cellular debris. The protein concentration was determined by BCA kit (Beyotime). Briefly, 50 μg of total protein was subjected to 10% SDS-PAGE gels and transferred to PVDF Transfer Membranes (Millipore, Billerica, MA, USA) at 100 V for 40–100 min. Afterwards, membranes were blocked for 3 h in 5% non-fat dry milk in TBST at room temperature and incubated with primary antibodies specific to Col1 (1:1000, Abcam, Cambridge, UK), Col3 (1:1000, Abcam, Cambridge, UK), α-SMA (1:1000, CST, USA), CD9, CD63 (1:1000, Proteintech, China), Smad2/3 Antibody Sampler Kit (1:1000, CST), IL-17RA (1:1000, Abcam, Cambridge, UK), SIP1 (1:1000, Abcam, Cambridge, UK), and β-actin (1:1000, Zhuangzhi, Xi′an) at 4 °C overnight. The next day, the membranes were incubated with HRP-conjugated anti-rabbit IgG secondary antibodies (1:3000, Boster, Wuhan, China) at 37 °C for 1 h. For chemiluminescence detection of proteins, immunoreactive traces on the membrane were visualized with ECL Kit (Millipore, USA) on a FluorChem FC system (Alpha Innotech), and the intensity of protein expression was analyzed by ImageJ software and normalized against β-actin.

### The wound scratch assays and trans-well assays

Approximately 100% confluent HSFs grown in 35-mm cell culture dishes were starved with serum-free medium for 12–16 h prior to stimulation with ADSC-Exo (20 μg/ml). Mitomycin C (MMC; 10 μg/ml, Invitrogen, Waltham, MA, USA) was supposed to totally inhibit cell proliferation. The monolayer was scratched with a 200 μl sterile pipette tip to create a wound gap, washed with PBS four times, and treated with ADSC-Exo or an equal volume of PBS. The distance between the scratch borders was measured by Image-Pro Plus 6.0 software at 4 points along the scratch after 24 h.

The upper chamber of a 24-well trans-well plate with a 8-μm aperture of the filter membrane (Corning, NY) was filled with 500 μl of complete medium containing FBS depleted of exosomes, and cell suspension of HSFs was seeded at a density of 5 × 10^4^/well. In total, 500 μl of culture medium supplemented with ADSC-Exo (20 μg/ml) or an equal volume of PBS was added to the lower chamber and incubated for 24 h. Then, HSFs were fixed with 4% paraformaldehyde for 30 min and washed with PBS three times. HSFs were dyed with 0.5% crystal violet staining solution (500 μl, Boster) and incubated for 30 min at room temperature. After washed with PBS, the number of migrated cells was observed under a microscope (FSX100, Olympus, Tokyo, Japan).

### The effect of ADSC-Exo on the proliferation of HSFs measured by flow cytometry

HSFs stimulated with ADSC-Exo or PBS in six-well cell culture plates were digested with 0.25% trypsin and subjected to centrifuge at 1000 rpm for 5 min. Then, the pellets were washed with PBS, cautiously added, dropwise with precooled 75% ethanol to make HSFs be fixed uniformly. HSFs were cryopreserved at − 20 °C at least for 2 h. Thereafter, HSFs were washed with PBS twice at 1500 rpm for 10 min, resuspended with 200 μl of PI/Rnase staining (BD, Biosciences), and incubated in the dark places for 15 min at room temperature. The percentage of cell cycle in each phase was detected by using BD Accuri™ C_6_ flow cytometer.

### Immunofluorescence staining

HSFs were cultured on 35-mm culture dishes with 14 mm glass diameter to approximately 50% confluence. HSFs exposed to ADSC-Exo or IL-17RA siRNA for 24 h were fixed with 4% paraformaldehyde at room temperature for 30 min. Cells were washed with PBS three times, permeated with 0.1% Triton X-100 in PBS for 30 min, and blocked in 2% BSA in PBS for 1 h. Primary antibodies (α-SMA, 1:200, CST, ki67, 1:200, CST, IL-17RA 1:200, Abcam) were diluted in 2% BSA and incubated overnight at 4 °C. The next day, HSFs were incubated with the secondary Cy3 antibody anti-rabbit (1:200) for 1 h at 37 °C and counterstained with DAPI, and the images were obtained by an Olympus FSX100 microscope.

### Luciferase reporter assay

To ensure that IL-17RA was indeed a direct target of miR-192-5p, we obtained luciferase-3′-untranslated region (3′UTR) reporter constructs of IL-17RA mRNA. Co-transfections of wild-type IL-17RA 3′UTR, mutant IL-17RA 3′UTR, or their non-targeting control RNA with miR-192-5p mimics at a final concentration of 50 nM were accomplished with lipofectamine 2000 transfection reagent. The samples were harvested after 24 h for luciferase assays (Promega, WI, USA).

### The effect of ADSC-Exo on in vivo experiment

Six- to eight-week-old male BABL/c mice were purchased from Experimental Animal Center of Fourth Military Medical University. The animal experimental protocols were performed in strict accordance with Experimental Animal Committee of Fourth Military Medical University (Xi′an, China). The mice were randomly divided into two groups: PBS or ADSC-Exo groups (70 μg diluted in 100 μl PBS); EGFP-NC or mIL17-RA shRNA-EGFP groups (HANBIO). In brief, the mice were anesthetized by isoflurane, 1 × 1 cm^2^ full-thickness defects were created on the dorsal skin. Three days later (day 3), ADSC-Exo or an equal volume of PBS (1 × 10^9^/pfu of Lv-IL-17RA or NC) was administered by subcutaneous injection into the wound using a 27-gauge needle for the consecutive 5 days. Digital photographs of wounds were obtained on days 3, 5, 7, 10, and 14. After 2 weeks, mice were sacrificed and wound tissues were harvested for the following histological analysis. There were six mice at least in each experimental group (*n* = 6).

### Histopathology analysis

The samples were fixed with 4% paraformaldehyde, dehydrated in graded ethanol, embedded in paraffin, and then cut into 5-μm-thick sections. H&E and Masson’s trichrome staining were used to detect the histological change and collagen deposition. For immunohistochemistry staining, the sections were immersed in 3% H_2_O_2_ after deparaffinization to eliminate the activity of endogenous peroxidase at 37 °C for 15 min and blocked with 5% BSA in PBS for 1 h to exclude the non-specific binding. Then, the slides were incubated with the primary antibodies against α-SMA and IL-17RA overnight at 4 °C. The next day, the slides were incubated with a PV6000 Histostain™ kit (ZSGB, Beijing, China) and stained with diaminobenzidine (ZSGB, Beijing, China). Images were obtained by FSX100 Bio Imaging Navigator (Olympus, Tokyo, Japan).

### Statistical analysis

All data were analyzed using SPSS17.0 software. Every experiment was repeated three times at least, and the data were shown as mean ± standard error of the mean. Student’s *t* test was used for the comparisons between two groups and analysis of variance (ANOVA) was used for multi-group comparisons. *p < 0.05* was considered statistically significant.

## Results

### The characterization of human ADSCs and ADSC-derived exosome

**Fig. 1:**
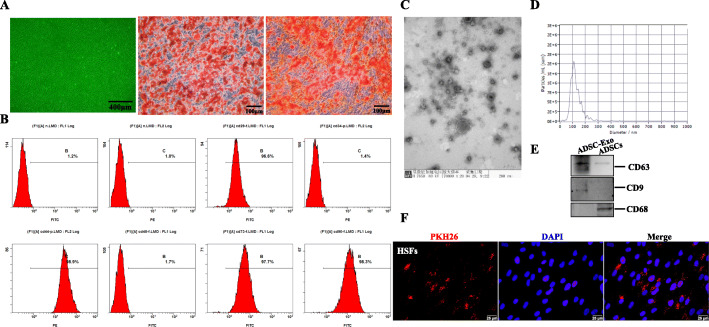
The identification of ADSCs and ADSC-Exo. **a** Optical morphology of human ADSCs under light field microscope, scale bars = 400 μm. Adipogenic and osteogenic differentiations measured by Oil Red O staining and Alizarin Red S staining, scale bars = 100 μm. **b** Representative flow cytometry analysis of ADSCs, the expression of mesenchymal stem cell surface markers (CD29 96.6%, CD44 98.9%, CD73 97.7%, CD90 98.3%) and hematopoietic stem cell surface markers (CD34 1.4%, CD45 1.7%). **c** The morphology of ADSC-Exo analyzed by TEM, scale bar = 200 nm. **d** The particle size distribution of ADSC-Exo measured by NTA. **e** Immunoblot analysis of known exosomal markers (CD9, CD63) and negative markers (CD68). **f** Representative images of the internalization of PKH-26-labeled ADSC-Exo into HSFs, scale bars = 25 μm

ADSCs displayed a remarkable fibroblast-like morphology and had the ability of multiple differentiation potential. Adipogenic differentiation exhibited lipid droplets in the cytoplasm and osteogenic differentiation indicated the presence of calcium deposition, as evidenced by Oil Red O staining and Alizarin Red S staining, respectively (Fig. 1a). Besides, ADSCs were highly positive for MSC (mesenchymal stem cells) surface markers, including CD29 (FITC 96.6%), CD44 (PE 98.9%), CD73 (FITC 97.7%), and CD90 (FITC 98.3%), but negative for HSC (hematopoietic stem cell) surface markers (CD34 PE 1.4% and CD45 FITC 1.7%) by flow cytometry analysis (Fig. 1b). The features demonstrated the isolated cells were consistent with typical ADSC characteristics. Furthermore, we collected cell-conditioned medium of ADSCs and extracted exosomes. As shown in Fig. 1c, ADSC-Exo presented a cup- or sphere-shaped morphology by TEM, NTA analysis identified the mean diameters of exosome was 113.6 nm (Fig. 1d), and immunoblotting was performed to confirm the presence of known exosomal markers (CD63 and CD9, Fig. 1e). The data indicated that the nanoparticles were consistent with the defined exosomes.

### ADSC-Exo inhibited the proliferation and migration of HSFs and alleviated the expression of pro-fibrotic markers in HSFs

**Fig. 2:**
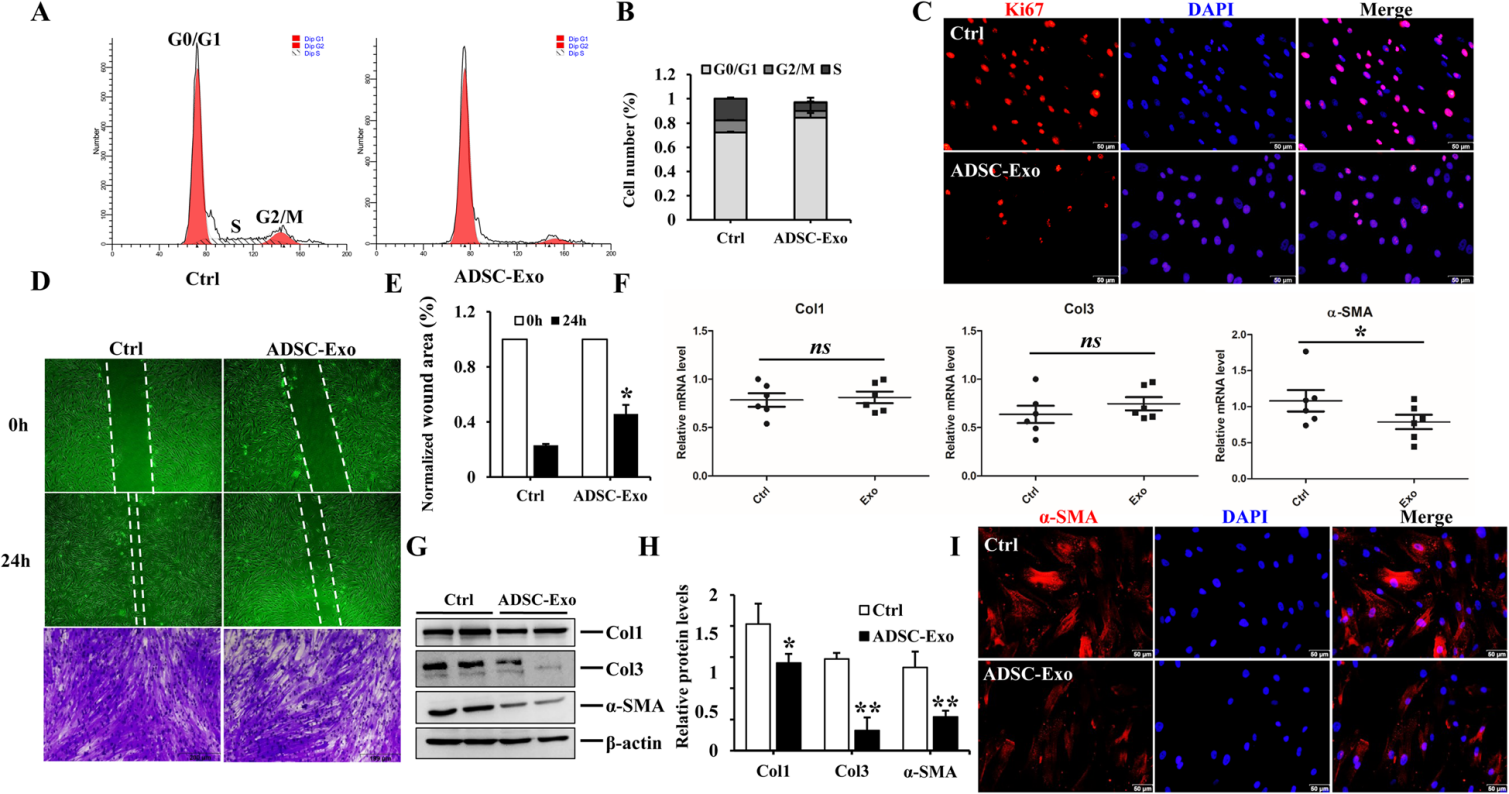
The effect of ADSC-Exo on HSFs. **a, b** Cell cycle analysis of HSFs exposed to ADSC-Exo or PBS. **c** Representative images of immunofluorescence staining of Ki67 in HSF exposure to ADSC-Exo or PBS, scale bar = 50 μm. **d, e** The effect of ADSC-Exo on HSF migration evaluated by the scratch wound assays and crystal violet staining, graphs showed the comparison of relative wound areas between ADSC-Exo and control group at 0 h and 24 h, original magnification (× 4 or × 10). **f** qRT-PCR analysis of the profibrotic factors in HSFs treated with ADSC-Exo or PBS, graph represented the expression of Col1, Col3, and α-SMA relative to that of GAPDH. **g, h** Immunoblot analysis of Col1, Col3, and α-SMA in HSFs stimulated with ADSC-Exo or PBS, the histogram demonstrated the relative band density to β-actin. **i** Representative images of α-SMA immunofluorescence staining in HSFs stimulated with ADSC-Exo or PBS, scale bar = 50 μm. The data was shown as mean ± SEM (*p < 0.05, **p < 0.01, ns: no differences)

Since we have proved that ADSC-CM (ADSCs derived conditional medium) could suppress hypertrophic scar fibrosis [30], as an essential and executive component of ADSC-CM, we hypothesized exosome played an important role in ameliorating hypertrophic scar fibrosis. To evaluate the effect of ADSC-Exo on HSFs, we firstly detected whether ADSC-Exo could be internalized into HSFs. As shown in Fig. 1f, PKH-26-labled ADSC-Exo were traced in the perinuclear and nuclear region of HSFs. We further found ADSC-Exo could inhibit the proliferation and migration of HSFs after stimulation for 24 h. For proliferation assays, images of immunofluorescence staining of Ki67 in HSFs treated with ADSC-Exo had a fluorescence intensity lower than that of the control group, and flow cytometry analysis also demonstrated ADSC-Exo could prolongate G0/G1 phase and shorten S phase to inhibit HSF proliferation (Fig. 2a–c). For migratory assays, the results showed the migration of HSFs was markedly inhibited in response to ADSC-Exo stimulation, as evidenced by the wound scratch assays and trans-well migration assays, there were statistical differences for the relative scratch areas between ADSC-Exo and control group (*p* < 0.05) (Fig. 2d, e). Besides, ADSC-Exo could downregulate the protein expression of Col1, Col3, and α-SMA in HSFs (Fig. 2g, h), there were significantly statistical differences compared to the control group (*p < 0.05*), whereas there was no noticeable change for the mRNA expression of collagen between two groups (Fig. 2f). The mRNA level and the immunofluorescence intensity of α-SMA were decreased in HSF exposure to ADSC-Exo (Fig. 2i), suggesting that ADSC-Exo could reverse the trans-differentiation of fibroblasts to myofibroblasts to alleviate hypertrophic scar fibrosis. These findings indicated that ADSC-Exo inhibited the biological function of HSFs to attenuate the fibrosis.

### ADSC-Exo accelerated wound healing and decreased collagen deposition in excisional model of BALB/c mice

**Fig. 3:**
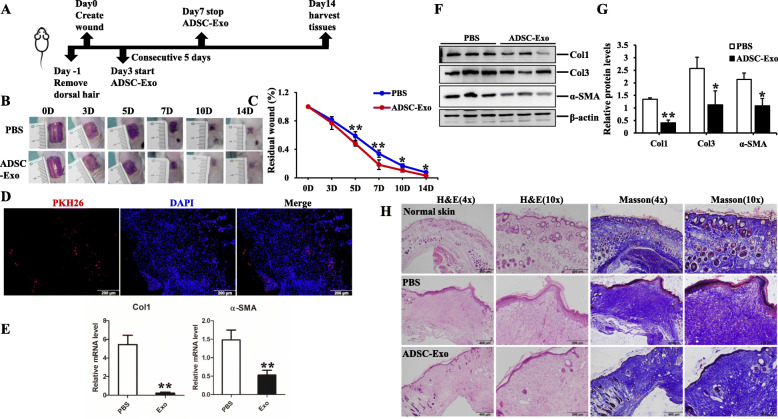
The effect of ADSC-Exo on the excisional model of BALB/c mice. **a** Schematic diagram of the experimental processes. **b, c** Digital photographs of wound area treated with ADSC-Exo or PBS post-wounding on day 3, 5, 7, 10, and 14. Histogram showed the relative distances analyzed by Image-Pro Plus. **d** The trace of PKH26-labeled ADSC-Exo in the wound of BALB/c mice on day 5, scale bars = 200 μm. **e** qRT-PCR analysis of Col1 and α-SMA in wound tissues of BALB/c mice treated with PBS or ADSC-Exo, graph represented the expression of Col1 and α-SMA relative to that of GAPDH. **f, g** Immunoblot analysis of Col1, Col3, and α-SMA in wound tissues of BALB/c mice treated with PBS or ADSC-Exo, histogram showed the relative band density to β-actin. **h** Representative images of H&E and Masson’s trichrome staining of the sections on day 14 post-wounding in normal skin, wound tissues treated with PBS or ADSC-Exo groups, scale bar = 400 μm, 100 μm. The data was shown as mean ± SEM (*p < 0.05, **p < 0.01)

The major challenge of animal model for hypertrophic scar is the differences in the structure of skin and subcutaneous tissue between human and animals [31, 32]. However, many animal models, such as mice, rats, rabbits, pigs, and other animals, are used for studies on hypertrophic scar formation. Unfortunately, none of the currently used animal models is ideal (summarized in Table 2 of supplementary materials) [33]. Mice are the most widely used species, and excisional full-thickness wounds are the widely used models in studying wound healing and hypertrophic scar, which is partly due to the availability of a large variety of mouse-specific biochemical reagents and transgenic strains compared to other animals [34, 35]. In the study, we focused on the structure and arrangement of collagen. To elucidate the effect of ADSC-Exo on animal model, we created the full-thickness defects on the dorsal skin of BALB/c mice, followed by subcutaneous injection of ADSC-Exo or an equal volume of PBS. Digital photograph showed ADSC-Exo significantly facilitated wound healing, as determined by smaller wound areas measured on day 5, 7, 10, and 14 post-wounding, and there were statistical differences for the comparison of wound areas on day 5, 7, 10, and 14 to that of day 0 (*p <* 0.05) (Fig. 3a, b). We traced the distribution of PKH26-labeled ADSC-Exo in wound tissues on day 5 (Fig. 3c) and harvested the samples on day 14 to analyze the histological feature and the fibrous protein expression. As shown in Fig. 3d–f, ADSC-Exo could significantly decrease the expression of Col1, Col3, and α-SMA to inhibit the fibrosis in wound tissues, and there were significantly statistical differences between two groups (*p* < 0.05). H&E and Masson’s trichrome staining were carried out to evaluate the extent of re-epithelialization and collagen deposition, as shown in Fig. 3g, and faster wound healing, less collagen deposition, and thinner, orderly arranged collagen structure were observed in ADSC-Exo-treated group. The in vivo study further confirmed the anti-fibrotic properties of ADSC-Exo and the potential therapeutic strategy for clinical treatment.

### MiR-192-5p in ADSC-Exo exerted the anti-fibrotic effect in HSFs

**Fig. 4:**
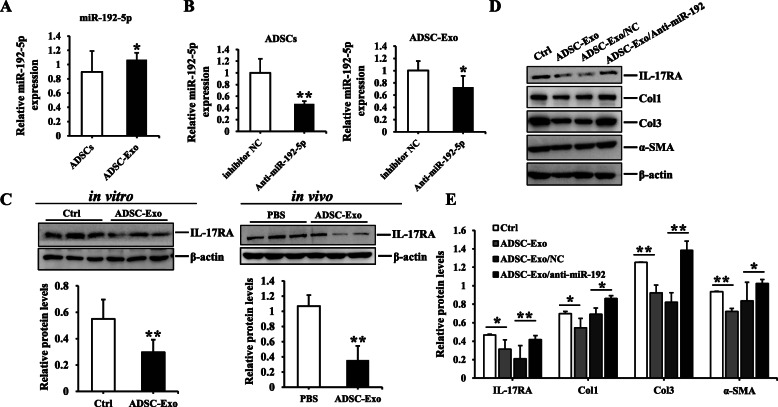
The effect of ADSC-exosomal miR-192-5p on HSFs. **a** The expression of miR-192-5p in ADSCs and ADSC-Exo. **b** qRT-PCR analysis of miR-192-5p in ADSCs transfected with miR-192-5p inhibitor NC or anti-miR-192 and their corresponding ADSC-Exo. **c** Immunoblot analysis of IL-17RA in HSFs and wound tissues of BALB/c mice exposure to ADSC-Exo or PBS. **d** Immunoblot analysis of IL-17RA, Col1, Col3, and α-SMA in HSFs stimulated with PBS, ADSC-Exo, ADSC-Exo/anti-miR-192-5p NC, or ADSC-Exo/anti-miR-192-5p, graph showed the relative band density to β-actin. Every experiment was repeated at least three times, and the data was shown as mean ± SEM (*p < 0.05; **p < 0.01)

The following problem was to investigate the exact mechanism underlying the anti-fibrotic properties of ADSC-Exo. It is well-known that microRNAs play the crucial role in improving skin fibrosis and promoting tissue regeneration, and then exosome could regulate the biological processes and cell-to-cell communication by miRNAs [10–12]. Therefore, we focused on the effect of miR-192-5p on hypertrophic scar fibrosis by literatures review [13, 14]. Interestingly, we found the expression of miR-192-5p in ADSC-Exo was higher than that of ADSCs (Fig. 4a). Subsequently, we transfected miR-192-5p inhibitor to silence miR-192-5p in ADSCs and collected cell-conditioned medium to isolate exosome. The results showed the expression of miR-192-5p was decreased in ADSCs transfected with miR-192-5p inhibitor and the corresponding exosome (ADSC-Exo/anti-miR-192-5p), and there were statistical differences compared to their negative controls (*p < 0.05*) (Fig. 4b). Furthermore, IL-17RA had been recognized as a driver of fibrosis [36–38], and we found ADSC-Exo could downregulate the expression of IL-17RA in HSFs and wound tissues of BALB/c mice (Fig. 4c). More importantly, the expression of IL-17RA, Col1, Col3, and α-SMA in HSF exposure to ADSC-Exo/anti-miR-192-5p were enhanced compared to ADSC-Exo/anti-miR-192-5p NC group (Fig. 4b), and there were remarkably statistical differences between two groups (*p* < 0.05) (Fig. 4d, e). These findings conclusively demonstrated that the anti-fibrotic effect of ADSC-derived exosomal miR-192-5p on HSFs had been closely linked to IL-17RA, so what was the regulatory relationship between miR-192-5p and IL-17RA in the formation of hypertrophic scar?

### MiR-192-5p directly targeted IL-17RA to suppress hypertrophic scar fibrosis

**Fig. 5:**
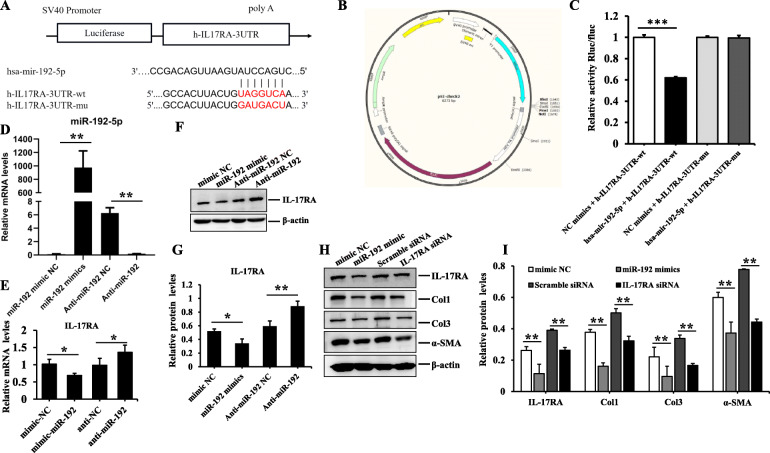
The verification of targeted regulatory relationship between miR-192-5p and IL-17RA in HSFs. **a** The binding sites predicted by bioinformatics algorithms. **b, c** The targeted modulation measured by luciferase reporter gene assays. **d** qRT-PCR analysis of miR-192-5p in HSFs transfected with mimics, inhibitors, and their corresponding negative control. **e–g** The expression of IL-17RA in HSFs transfected with mimics, inhibitors, and their corresponding negative control examined by qRT-PCR and WB. **h, i** Immunoblotting analysis of the protein expression of IL-17RA, Col1, Col3, and α-SMA in HSFs exposed to miR-192-5p mimics/mimic NC, IL-17RA siRNA/scramble siRNA, graphs showed their levels relative to that of β-actin. Every experiment was repeated at least three times, the data was shown as mean ± SEM (*p < 0.05, **p < 0.01, ***p < 0.001)

MicroRNAs modulate gene expression post-transcriptionally by binding to 3′UTR of target mRNAs to affect translational repression. Bioinformatics algorithm (Target Scan 7.0, PicTar and miRanda) analysis suggested that miR-192-5p contained the consequential pairing of target region positions 2012–2019 of IL-17RA 3′-UTR (Fig. 5a). Luciferase reporter assay was performed to confirm the target relationship, miR-192-5p mimic significantly reduced the luciferase activity of HEK293 cells transfected with reporter plasmid containing a wild-type 3′-UTR sequence of IL-17RA, and there were distinctly statistical differences compared to the negative control of miR-192-5p mimics (*p* < 0.001), whereas that of mutant IL-17RA 3′UTR did not appear to be suppressed, indicating no binding (Fig. 5b, c). Subsequently, HSFs were transfected with miR-192-5p mimics or inhibitor and their corresponding negative control using lipo2000 (Fig. 5d), and qPCR analysis was performed to determine the expression of miR-192-5p and IL-17RA. We further found the expression of IL-17RA was downregulated in HSFs transfected with miR-192-5p mimics, but its inhibitor had the opposite effect (Fig. 5e–g). Meanwhile, the expression of collagen and α-SMA were also reduced in HSFs transfected with IL-17RA siRNA, which was in accordance with the effect of miR-192-5p mimics on HSFs, and there were significantly statistical differences compared to their negative controls (*p* < 0.05) (Fig. 5h, i). The aforementioned data suggested that IL-17RA was a direct target of miR-192-5p in hypertrophic scar fibrosis.

### The expression of IL-17RA in HS

**Fig. 6:**
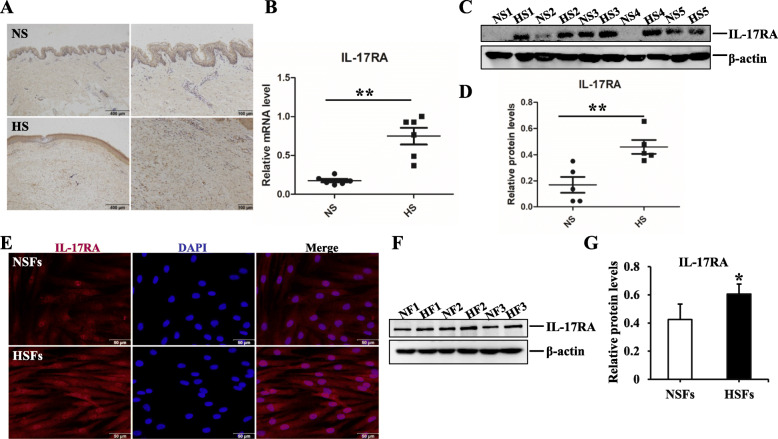
The expression of IL-17RA in NS, HS, and their derived fibroblasts (NSFs and HSFs). **a** Representative images of IL-17RA in NS and HS detected by immunohistochemistry staining, scale bar = 400 μm and 100 μm. **b** qRT-PCR analysis of IL-17RA in NS and HS tissues. **c, d** Immunoblot analysis of IL-17RA in NS and HS tissues. **e** Representative images of immunofluorescence staining of IL-17RA in NSFs and HSFs, scale bar = 50 μm. **f** Immunoblot analysis of IL-17RA in NSFs and HSFs. Every experiment was repeated at least three times, the data was shown as mean ± SEM (*p < 0.05; **p < 0.01)

To explore the role of IL-17RA in scar formation, we investigated its expression in NS, HS, and their derived fibroblasts (NSFs and HSFs). For histological detection, as shown in Fig. 6a, there were more positive IL-17RA+ fibroblasts in the dermis of hypertrophic scar tissues by immunohistochemistry staining. Besides, IL-17RA was overexpressed in HS both in genetic level and in protein level, there were significantly statistical differences between NS and HS (*p* < 0.05) (Fig. 6b–d). For cytological detection, immunofluorescence staining demonstrated there were more IL-17RA+ fluorescence intensity in HSFs and immunoblot assays were carried out to assess the protein level of IL-17RA, the result indicated IL-17RA was highly expressed in HSFs, which was in line with the histological findings (Fig. 6e–g). These findings suggested IL-17RA had been strongly associated with hypertrophic scar fibrosis.

### IL-17RA silence exerted the anti-fibrotic properties in HSFs and BALB/c excisional model

**Fig. 7:**
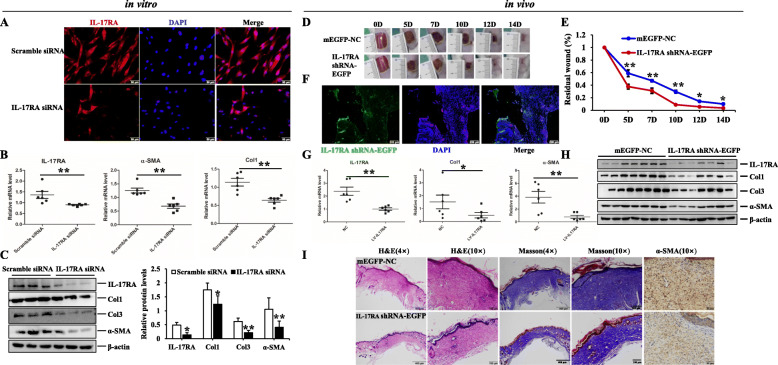
The effect of IL-17RA silence on HSFs and excisional model of BALB/c mice. **a** The efficiency of IL-17RA siRNA in HSFs examined by immunofluorescence staining, scale bar = 50 μm. **b** qRT-PCR analysis of IL-17RA, Col1, and α-SMA in HSFs treated with IL-17RA siRNA or scramble siRNA. **c** Immunoblot analysis of IL-17RA, Col1, Col3, and α-SMA in HSFs exposed to IL-17RA siRNA or scramble siRNA, graphs showed the relative band density to β-actin. **d, e** The morphology of wound healing observed in different periods. Line chart showed the relative wound areas. **f** The trace of Lv-IL-17RA in wound tissues on day 5 detected by frozen sections, scale bar = 200 μm. **g** qRT-PCR analysis of IL-17RA, Col1, and α-SMA in wound tissues of BALB/c mice stimulated with mEGFP-NC or IL-17RA shRNA-EGFP, the graph represented their expression relative to that of GAPDH. **h** Immunoblot analysis of the aforementioned molecules. **i** Representative images showed the histopathological change, collagen deposition, and positive α-SMA+ fibroblasts determined by H&E, Masson’s trichrome, and immunohistochemistry staining, respectively, scale bar = 400 μm, 100 μm, 50 μm. The data was shown as mean ± SEM (*p < 0.05; **p < 0.01)

Since the expression of IL-17RA was upregulated in HS, we would subsequently explore the effect of IL-17RA silence on HSFs and animal model. Firstly, we verified the efficiency of IL-17RA siRNA in HSFs by immunofluorescent staining (Fig. 7a). Then, the expression of Col1, Col3, and α-SMA were prominently downregulated in IL-17RA silence-induced HSFs, and there were significantly statistical differences compared to the control group (*p* < 0.05) (Fig. 7b, c). Secondly, we traced EGFP-labeled IL-17RA shRNA in wound tissues on day 5 (Fig. 7f) and found IL-17RA silence could accelerate wound closure, and there were smaller wound areas on day 5, 7, 10, 12, and 14 in BALB/c mice treated with Lv-IL-17RA (Fig. 7d, e). In addition, the mRNA and protein levels of Col1, Col3, and α-SMA were remarkably reduced in wound tissues of BALB/c mice treated with Lv-IL-17RA, and there were statistical differences between EGFP-NC group and Lv-IL-17RA group (*p* < 0.05) (Fig. 7g, h). Furthermore, H&E and Masson’s trichrome staining were performed to examine the histological change and collagen arrangement, the result indicated that the mice in IL-17RA silence group manifested faster wound healing, less collagen deposition, thinner and arranged orderly collagen structure, and less positive α-SMA+ fibroblasts of dermis (Fig. 7i). These findings demonstrated IL-17RA silence obviously ameliorated collagen synthesis and myofibroblasts activation, and then exerted the anti-fibrotic properties and potential therapeutic effect for HS fibrosis.

### ADSC-Exo and IL-17RA silence had the similar effect on Smad signaling pathway in HSFs

**Fig. 8:**
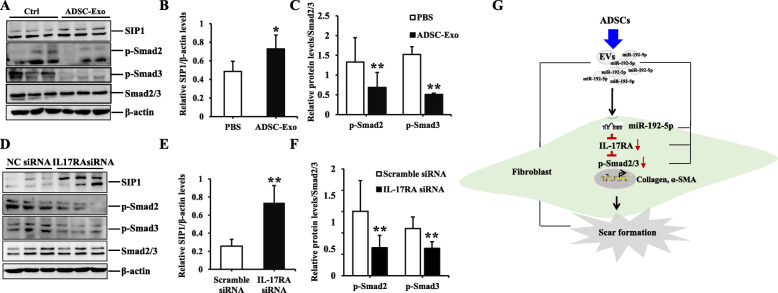
The effect of ADSC-Exo or IL-17RA siRNA on Smad pathway in HSFs. **a–c** Immunoblot analysis of SIP1, p-Smad2, and p-Smad3 in HSFs stimulated with ADSC-Exo or PBS, graphs showed the relative band density to β-actin or total Smad2/3, respectively. The data was shown as mean ± SEM (*p < 0.05; **p < 0.01). **d–f** Immunoblot analysis of SIP1, p-Smad2, and p-Smad3 in HSFs treated with IL-17RA siRNA or scramble siRNA, graph showed the relative band density to β-actin or total Smad2/3, respectively. The data was shown as mean ± SEM (**p < 0.01). **g** Schematic diagram of the experimental results

It is well-known that Smad signaling pathway is involved with almost all of fibrotic diseases, so we would investigate the effect of ADSC-Exo and IL-17RA silence on Smad pathway in HSFs. The results indicated ADSC-Exo and IL-17RA silence could increase the expression of SIP1 and decrease the expression of p-Smad2 or p-Smad3 (Fig. 8a, d), suggesting that ADSC-Exo or IL-17RA silence could regulate the Smad signal transduction to inhibit the fibrosis in HSFs, and there were statistical differences between two groups (ADSC-Exo/PBS or IL-17RA siRNA/scramble siRNA, *p* < 0.05) (Fig. 8b, c, e, f). These findings further clarified the anti-fibrotic effect of ADSC-Exo was realized by miR-192-5p/IL-17RA/Smad axis (summarized in Fig. 8g).

## Discussion

The study provides the following major findings: Firstly, in opposite to some prior studies demonstrating ADSC-Exo facilitated the proliferation, migration of fibroblasts, and collagen synthesis [39], we verified ADSC-Exo could inhibit the bioactivity of HSFs and collagen deposition to play the anti-fibrotic role in the formation of hypertrophic scar. The discrepancy could be largely attributed to the type of fibroblasts: activated (HSFs) or nonactivated (NSFs), and we focused on the effect of ADSC-Exo on HSFs. Secondly, we demonstrated miR-192-5p in ADSC-Exo could impair the fibrosis in HSFs and IL-17RA was a direct target of miR-192-5p. Thirdly, IL-17RA modulated Smad pathway to inhibit the expression of pro-fibrotic proteins in HSFs. In summary, the results unveiled the molecular mechanism underlying the anti-fibrotic properties of ADSC-Exo were implemented by miR-192-5p/IL-17RA/Smad axis. The study displayed the sufficient evidences for the possible therapeutic effect of ADSC-Exo.

Hypertrophic scar, a severe fibrotic skin disease, has always been a clinical challenge to urgently solve. Fibroblasts isolated from the dermis of hypertrophic scar tissues remain persistently hyperactive and proliferative, resulting in myofibroblasts contractile and ECM synthesis [40, 41]. Consequently, we measured hypertrophic scar fibrosis by the expression of Col1, Col3, and α-SMA in the study. Hypertrophic scar could cause the destruction of skin architecture and the loss of joint function to impact the quality of patients’ life. To date, there were no effective therapeutic strategies for HS treatment. Therefore, the study was aimed to investigate a novel way for clinical application.

ADSCs had been identified to play the important role in alleviating the progress of fibrotic diseases [42–44], and our previous study also revealed ADSC-conditioned medium exerted the anti-fibrotic effect in the formation of hypertrophic scar [30]. Exosomes are 30-150 nm, small extracellular vesicles secreted into culture supernatants by almost all of cell types [45]. Exosomes could be a promising therapeutic strategy because of less immunogenic, non-cytotoxic, and non-mutagenic to the recipient compared with other gene delivery vehicles [46, 47]. Mesenchymal stem cell-derived exosomes facilitated wound healing and blocked myofibroblast differentiation in idiopathic pulmonary fibrosis [12, 48]. Besides, exosomes had emerged as important agents in liver injury and fibrosis progression [49]. In accordance with these findings, we demonstrated the anti-fibrotic properties of ADSC-Exo in the formation of hypertrophic scar. The results showed PKH26-labeled ADSC-Exo was internalized into HSFs and ADSC-Exo inhibited the proliferation, the migration, and the contraction of HSFs. In addition, ADSC-Exo decreased collagen production and myofibroblast activity in HSFs. More importantly, ADSC-Exo promoted wound healing and alleviated collagen deposition to suppress hypertrophic scar fibrosis in excisional model of BALB/c mice. As noted above, ADSC-Exo inhibited hypertrophic scar fibrosis by in vitro and in vivo experiments. However, the mechanism underlying the anti-fibrotic effect of ADSC-Exo had not been fully elucidated.

Emerging evidence had indicated exosomes, the paracrine signal mediators, impacted the progression of fibrotic diseases by transferring anti-fibrotic or pro-fibrotic miRNAs to target cells and affected pathological fibrogenesis [50]. One of the major challenges for synthetic miRNAs was quickly degraded by the high activity of ribonuclease in plasma, but exosome would protect miRNAs from degradation [51, 52]. It had been reported that mesenchymal stem cell-derived extracellular vesicles suppressed the proliferation of fibroblast by downregulating FZD6 expression in fibroblasts via microRNA-29b-3p in idiopathic pulmonary fibrosis [53]; other investigators had shown that macrophage-derived exosomes attenuated the fibrosis in airway epithelial cells through delivery of anti-fibrotic miR-142-3p [50]. Our study found miR-192-5p was highly expressed in ADSC-Exo and ADSC-exosomal miR-192-5p impaired the expression of pro-fibrotic proteins in HSFs. Meanwhile, exosomes derived from ADSCs transfected with miR-192-5p inhibitor (ADSC-Exo/anti-miR-192) could reverse the reduction of collagen and α-SMA expression to emphasize the importance of miR-192-5p in ADSC-Exo.

As a class of small endogenous noncoding RNAs, the major function of miRNAs is to downregulate the expression of target genes by binding to 3′UTR of target genes [54]. Bioinformatic analysis indicated miR-192-5p had the complementary sequences of IL-17RA 3′UTR, and luciferase reporter assays verified the targeted regulatory relationship between miR-192-5p and IL-17RA. As expected, the expression of IL-17RA was downregulated in HSFs transfected with miR-192-5p mimics, whereas IL-17RA level was increased in HSFs transfected with miR-192-5p inhibitor. Strikingly, the expression of Col1, Col3, and α-SMA in HSFs transfected with IL-17RA siRNA were decreased, which was similar with that of HSFs stimulated with miR-192-5p mimics. As mentioned above, the results suggested miR-192-5p directly targeted IL-17RA to regulate hypertrophic scar fibrosis. Therefore, we would investigate the effect of IL-17RA on scar formation in the following experiment.

IL-17A receptor (IL-17RA) is ubiquitously expressed on many cell types of human body and highly expressed in the bone marrow, thymus, and spleen [55]. In the airways, IL-17RA is mainly expressed on fibroblasts, epithelial cells, smooth muscle cells, and microvascular endothelial cells [21, 22]. However, the effect of IL-17RA on skin fibroblasts has not been elucidated. In the study, we explored the expression of IL-17RA in hypertrophic scar and found IL-17RA was overexpressed in HSFs, and then IL-17RA silence decreased the expression of collagen and α-SMA in HSFs. Meanwhile, IL-17RA silence facilitated wound healing, attenuated collagen deposition, and inhibited myofibroblast activity in excisional model of BALB/c mice. In line with our findings, it had been suggested that specific blockade of IL-17RA in the fibroblasts of hypertensive heart could inhibit collagen production and potentially ameliorate fibrosis [56]. Besides, neutralization of IL-17RA protected against adenoviral IL-1β-induced airway inflammation and fibrosis [25]. Moreover, IL-17RA deficiency largely protected mice from CCl_4_-induced liver fibrosis and inflammation-induced damage [57]. As described above, IL-17RA silence alleviated collagen deposition and myofibroblasts trans-differentiation to suppress HS fibrosis. Next, we explored the possible mechanism underlying the effect of IL-17RA on hypertrophic scar fibrosis.

It has been reported that hypertrophic scar is closely associated with Smad signaling pathway and Smads proteins are involved in signal transduction and transcriptional regulation [40, 58]*.* In canonical Smad pathway, phosphorylation of Smad2 and Smad3 bind to Smad4 and form Smad complex, which translocate into nucleus to regulate the transcription of target genes. Our previous study suggested the expression of p-Smad2/p-Smad3 were increased and the expression of SIP1 was decreased in HS and HSFs [59]. There were recently reported studies elucidating that human umbilical cord mesenchymal stem cell-derived exosomes (hucMSC-Exo) significantly reduced the expression of collagen and p-Smad2 to alleviate liver fibrosis in vivo [60]. Other than that, MSC-Exo regulated the fibrosis repair of injured endometrium by downregulating Smad signaling pathway, demonstrating that phosphorylation levels of Smad2 were gradually decreased with the increased concentration of exosome in BMSCs and Exo treatment groups [61]. Besides, levels of collagen, α-SMA, and phosphorylation of Smad2/3 were markedly decreased in fibroblasts treated with hucMSC-derived exosomes, suggesting that hucMSC-derived exosomes could inhibit dermal fibroblast-myofibroblast transition by inhibiting the Smad signaling pathway [62]. In line with these findings, we found the protein levels of p-Smad2/p-Smad3 were reduced in HSFs stimulated with ADSC-Exo, but the expression of SIP1 was increased in HSF exposure to ADSC-Exo in the study. Conversely, some investigators had shown that ADSC-Exo effectively upregulated the protein expression of p-Smad2/3 to attenuate ultraviolet B-mediated photoaging in human dermal fibroblasts [63]. The discrepancy had been attributed to the status of fibroblasts: quiescent condition or activated condition. To the best of our knowledge, no such association has ever been reported between IL-17RA and Smad signaling pathway in fibrotic diseases. However, the researchers found IL-17A/IL-17RA axis was necessary for the production of TGF-β, a critical mediator of tissue remodeling and collagen deposition in pulmonary fibrosis [56]. TGF-β was known to phosphorylate Smad proteins, such as Smad2 and Smad3 [64, 65]. In the study, we observed the expression of p-Smad2/p-Smad3 were downregulated and the expression of SIP1 was enhanced in HSFs stimulated with IL-17RA siRNA, whereas the levels of total Smad2/3 were unchanged. As mentioned above, these findings indicated ADSC-Exo or IL-17RA silence regulated Smad signal pathway to exert the anti-fibrotic effect in HSFs. Dramatically, there was evidence suggesting that miR-192 promoter had the complementary sequences of Smad [66], which might be another feasible mechanism associated with hypertrophic scar fibrosis. In consequence, we drew the preliminary conclusion that ADSC-Exo attenuated hypertrophic scar fibrosis by miR-192-5p/IL-17RA/Smad axis.

## Conclusions

In the study, ADSC-Exo was demonstrated to effectively inhibit the bioactivity, collagen deposition, and myofibroblasts trans-differentiation of HSFs, and then ADSC-Exo also facilitated wound healing and attenuated collagen synthesis in the excisional model of BALB/c mice. In addition, we verified miR-192-5p in ADSC-Exo could alleviate the fibrosis in HSFs and directly target IL-17RA to regulate Smad pathway in the formation of hypertrophic scar. Our study provided a novel therapeutic strategy and elucidated the special mechanism for clinical treatment of hypertrophic scar.

## Supplementary Information

**Additional file 1.**

## Data Availability

Not applicable.

## Abbreviations

HS: Hypertrophic scar
α-SMA: α-smooth muscle actin
Col1: Type I collagen
Col3: Type III collagen
ADSCs: Adipose tissues derived mesenchymal stem cells
ADSC-CM: ADSC-conditioned medium
ADSC-Exo: ADSC-derived exosome
DMEM: Dulbecco’s modified Eagle’s medium
FBS: Fetal bovine serum
siRNA: Small interfering RNA
DAPI: 4′,6-Diamidino-2-phenylindole
GAPDH: Glyceraldehyde-3-phosphate dehydrogenase
BSA: Bovine serum albumin
NS: Normal skin
HSFs: HS-derived fibroblasts
NSFs: NS-derived fibroblasts
TEM: Transmission electron microscope
NTA: Nanoparticle tracking analysis
3′UTR: 3′-Untranslated regions
SIP1: Smad interacting protein 1
