# Correction to: Exosomes derived from human adipose mesenchymal stem cells attenuate hypertrophic scar fibrosis by miR-192-5p/IL-17RA/Smad axis

**DOI:** 10.1186/s13287-021-02568-3

**Published:** 2021-09-03

**Authors:** Yan Li, Jian Zhang, Jihong Shi, Kaituo Liu, Xujie Wang, Yanhui Jia, Ting He, Kuo Shen, Yunchuan Wang, Jiaqi Liu, Wei Zhang, Hongtao Wang, Zhao Zheng, Dahai Hu

**Affiliations:** 1 Department of Burns and Cutaneous Surgery, Xijing Hospital, Fourth Military Medical University, 127 West Chang-le Road, Xi’an, 710032 Shaanxi China; 2 Department of Plastics and Aesthetic Surgery, The First Affiliated Hospital of Xi’an Medical University, No.48 West Fenghao Road, Xi’an, 710077 Shaanxi China

## Correction to: Stem Cell Research & Therapy (2021) 12:221 10.1186/s13287-021-02290-0

The original article [1] contained an error in author Hongtao Wang's name which has since been corrected.
