# Supplementary material for: Exosomes derived from human adipose mesenchymal stem cells attenuate hypertrophic scar fibrosis by miR-192-5p/IL-17RA/Smad axis
Source: Stem Cell Res Ther. 2021 Mar 31;12:221. doi: 10.1186/s13287-021-02290-0 (PMC8010995; doi:10.1186/s13287-021-02290-0)
Supplement: Supplementary file 1 — Additional file 1. [file 13287_2021_2290_MOESM1_ESM.docx]

The primer pairs used are listed in Table1.

| name | Forward | | Reverse |
| --- | --- | --- | --- |
| hCol1 | | 5′-GAGGGCAACAGCAGGTTCACTTA-3′ | 5′-TCAGCACCACCGATGTCCA-3′ |
| hCol3 | | 5′-CCACGGAAACACTGGTGGAC-3′ | 5′-GCCAGCTGCACATCAAGGAC-3′ |
| hα-SMA | | 5′-GACAATGGCTCTGGGCTCTGTAA-3′ | 5′-TGTGCTTCGTCACCCACGTA-3′ |
| hIL-17RA | | 5′- AGATCCTAGCACTGGGTCCACAC-3′ | 5′- TGGCACACTTCAAACCATGAGA-3′ |
| hGAPDH | | 5′-GCACCGTCAAGCTGAGAAC-3′ | 5′-TGGTGAAGACGCCAGTGGA-3′ |
| mα-SMA | | 5′- GACAATGGCTCTGGGCTCTGTA-3′ | 5′- TTTGGCCCATTCCAACCATTA-3′ |
| mCol1 | | 5′-TGCTTGCAGTAACTTCGTGCCTA-3′ | 5′-CATGGGACCATCAACACCATC-3′ |
| mIL-17RA | | 5′- ACAGCAGCCTTGTGGCGTAG-3′ | 5′-TTCCCACAGCATTTCATGGTC -3′ |
| mGAPDH | | 5′-AAATGGTGAAGGTCGGTGTGAAC-3′ | 5′-CAACAATCTCCACTTTGCCACTG-3′ |

| Human miR-192-5p | 5′-GACCTATGAATTGACAGCC-3′ |  |
| --- | --- | --- |
| U6 | 5′-CTCGCTTCGGCAGCACA-3′ |  |
| Universal reverse | 5′-GTGCAGGGTCCGAGGT-3′ |  |
| miR-192-5p mimic | 5′-CUGACCUAUGAAUUGACAGCC-3′ | 5′-CUGCCAAUUCCAUAGGUCACAG-3′ |
| Anti-miR-192-5p | 5′-GGCUGUCAAUUCAUAGGUCAG-3′ | 5′-CAUUAAUGUCGGACAACUCAAU-3′ |

Table 2 the analysis of different animal models for hypertrophic scar

| **Species** | **Model** | **Distinguish from human** | **Advantages** | **Disadvantages** |
| --- | --- | --- | --- | --- |
| Mice | Full thickness excisional model investigated by Galiano et al. [[1](file:///D:\2021-02-25-1\supplementary.docx#_ENREF_1)] and Jimi et al.[[2](file:///D:\2021-02-25-1\supplementary.docx#_ENREF_2)] | a subcutaneous muscle layer known as the “panniculus carnosus,” which in humans is largely vestigial [[3](file:///D:\2021-02-25-1\supplementary.docx#_ENREF_3)] | operate and handle readily, the availability of a large variety of mouse-specific biochemical reagents and transgenic animals, used widely. | minimal scarring due to the loose skin and low tension environment [[4](file:///D:\2021-02-25-1\supplementary.docx#_ENREF_4)] |
|  | Bleomycin-induced subcutaneous fibrosis [[5](file:///D:\2021-02-25-1\supplementary.docx#_ENREF_5)] | Scleroderma, a dermatological disease hardening of the dermis *via* accumulation of ECM proteins | Presenting the pathological features of skin thickening | The restriction of replicating the autoimmune nature of scleroderma and the progress of hypertrophic scar formation and the limitation of clinical relevance [[6](file:///D:\2021-02-25-1\supplementary.docx#_ENREF_6)] |
| Rats | Burn wound model [[7](file:///D:\2021-02-25-1\supplementary.docx#_ENREF_7)] or full thickness excisional model supplemented with a mechanical load device [[8](file:///D:\2021-02-25-1\supplementary.docx#_ENREF_8)] | Similar to mice, rats do not develop hypertrophic scar as the outcome of wound repair under normal conditions due to the strong contraction of skin post-wounding | loose-skinned animals heal wounds by muscle contraction [[9](file:///D:\2021-02-25-1\supplementary.docx#_ENREF_9)] | the availability of transgenic strains compared to mice is limited |
| Rabbits | Rabbit ear scar model proposed by Morris et al. in 1997 [[10](file:///D:\2021-02-25-1\supplementary.docx#_ENREF_10)] | full-thickness ischemic wounds of the skin and subdermal auricular perichondrium on the ventral side of the rabbit ears [[10](file:///D:\2021-02-25-1\supplementary.docx#_ENREF_10)] | the high reliability and reproducibility, scars formation visually similar to the human HS and [[11](file:///D:\2021-02-25-1\supplementary.docx#_ENREF_11)]. | lack of the availability of transgenic strains, which limits the application in studying the molecular mechanisms of hypertrophic scar and the extrapolation of the in vivo experimental finding to the humans [[12](file:///D:\2021-02-25-1\supplementary.docx#_ENREF_12)]. |
| Pigs | Full thickness defects first fully characterized by Gibran’s group[[13](file:///D:\2021-02-25-1\supplementary.docx#_ENREF_13)]. | the presence of subcutaneous fascia prevents skin contraction during wound closure and differentiates from other mammals. | The substantially similar anatomy and physiology to human skin compared to other animals and the fidelity [[14](file:///D:\2021-02-25-1\supplementary.docx#_ENREF_14)]. | the application is limited by high costs, labor intensity, difficulties in animal care, and relatively complicated ethical issues[[15](file:///D:\2021-02-25-1\supplementary.docx#_ENREF_15)]. |

**References:**

1. Galiano RD, Tepper OM, Pelo CR, Bhatt KA, Callaghan M, Bastidas N, Bunting S, Steinmetz HG, Gurtner GC: **Topical vascular endothelial growth factor accelerates diabetic wound healing through increased angiogenesis and by mobilizing and recruiting bone marrow-derived cells**. *The American journal of pathology* 2004, **164**(6):1935-1947.

2. Jimi S, De Francesco F, Ferraro GA, Riccio M, Hara S: **A Novel Skin Splint for Accurately Mapping Dermal Remodeling and Epithelialization During Wound Healing**. *Journal of cellular physiology* 2017, **232**(6):1225-1232.

3. Rhea L, Dunnwald M: **Murine Excisional Wound Healing Model and Histological Morphometric Wound Analysis**. *Journal of visualized experiments : JoVE* 2020(162).

4. Aarabi S, Longaker MT, Gurtner GC: **Hypertrophic scar formation following burns and trauma: new approaches to treatment**. *PLoS medicine* 2007, **4**(9):e234.

5. Willenborg S, Eckes B, Brinckmann J, Krieg T, Waisman A, Hartmann K, Roers A, Eming SA: **Genetic ablation of mast cells redefines the role of mast cells in skin wound healing and bleomycin-induced fibrosis**. *The Journal of investigative dermatology* 2014, **134**(7):2005-2015.

6. Padmanabhan J, Maan ZN, Kwon SH, Kosaraju R, Bonham CA, Gurtner GC: **In Vivo Models for the Study of Fibrosis**. *Advances in wound care* 2019, **8**(12):645-654.

7. Golberg A, Villiger M, Khan S, Quinn KP, Lo WCY, Bouma BE, Mihm MC, Jr., Austen WG, Jr., Yarmush ML: **Preventing Scars after Injury with Partial Irreversible Electroporation**. *The Journal of investigative dermatology* 2016, **136**(11):2297-2304.

8. Murphy A, LeVatte T, Boudreau C, Midgen C, Gratzer P, Marshall J, Bezuhly M: **Angiotensin II Type I Receptor Blockade Is Associated with Decreased Cutaneous Scar Formation in a Rat Model**. *Plastic and reconstructive surgery* 2019, **144**(5):803e-813e.

9. Li J, Wang J, Wang Z, Xia Y, Zhou M, Zhong A, Sun J: **Experimental models for cutaneous hypertrophic scar research**. *Wound repair and regeneration : official publication of the Wound Healing Society [and] the European Tissue Repair Society* 2020, **28**(1):126-144.

10. Morris DE, Wu L, Zhao LL, Bolton L, Roth SI, Ladin DA, Mustoe TA: **Acute and chronic animal models for excessive dermal scarring: quantitative studies**. *Plastic and reconstructive surgery* 1997, **100**(3):674-681.

11. Yagmur C, Guneren E, Kefeli M, Ogawa R: **The effect of surgical denervation on prevention of excessive dermal scarring: a study on rabbit ear hypertrophic scar model**. *Journal of plastic, reconstructive & aesthetic surgery : JPRAS* 2011, **64**(10):1359-1365.

12. van den Broek LJ, Limandjaja GC, Niessen FB, Gibbs S: **Human hypertrophic and keloid scar models: principles, limitations and future challenges from a tissue engineering perspective**. *Experimental dermatology* 2014, **23**(6):382-386.

13. Zhu KQ, Engrav LH, Tamura RN, Cole JA, Muangman P, Carrougher GJ, Gibran NS: **Further similarities between cutaneous scarring in the female, red Duroc pig and human hypertrophic scarring**. *Burns : journal of the International Society for Burn Injuries* 2004, **30**(6):518-530.

14. Sullivan TP, Eaglstein WH, Davis SC, Mertz P: **The pig as a model for human wound healing**. *Wound repair and regeneration : official publication of the Wound Healing Society [and] the European Tissue Repair Society* 2001, **9**(2):66-76.

15. Foubert P, Zafra D, Liu M, Rajoria R, Gutierrez D, Tenenhaus M, Fraser JK: **Autologous adipose-derived regenerative cell therapy modulates development of hypertrophic scarring in a red Duroc porcine model**. *Stem Cell Res Ther* 2017, **8**(1):261.
